# Supplementary material for: The impact of the early COVID-19 pandemic on maternal mental health during pregnancy and postpartum
Source: PLoS One. 2024 Sep 20;19(9):e0310902. doi: 10.1371/journal.pone.0310902 (PMC11414898; doi:10.1371/journal.pone.0310902)
Supplement: S1 Table — (PDF) [file pone.0310902.s001.pdf]

study (1=pre-pandemic; 2=pandemic)	EPDS_T1	EPDS_T2a	EPDS_T2b	EPDS_T3	STAI_Trait_T2a	STAI_Trait_T2b	STAI_Trait_T3	PBQ_T2a	PBQ_T2b	PBQ_T3	LIPS_T2a	LIPS_T2b	LIPS_T3
1.00	0	99.00	99.00	99.00	99.00	99.00	99.00	99.00	99.00	99.00	99.00	99.00	99.00
1.00	7	7.00	7.00	99.00	33.00	33.00	99.00	3.00	3.00	99.00	116.00	116.00	99.00
1.00	2	99.00	99.00	99.00	99.00	99.00	99.00	99.00	99.00	99.00	99.00	99.00	99.00
1.00	8	5.00	5.00	6.00	32.00	32.00	32.00	6.00	6.00	4.00	126.00	126.00	99.00
1.00	8	99.00	99.00	99.00	99.00	99.00	99.00	99.00	99.00	99.00	99.00	99.00	99.00
1.00	5	2.00	2.00	5.00	30.00	30.00	33.00	9.00	9.00	4.00	114.00	114.00	123.00
1.00	5	17.00	17.00	99.00	41.00	41.00	99.00	12.00	12.00	99.00	107.00	107.00	99.00
1.00	11	1.00	1.00	12.00	33.00	33.00	44.00	6.00	6.00	20.00	115.00	115.00	111.00
1.00	13	7.00	7.00	99.00	28.00	28.00	99.00	12.00	12.00	99.00	119.00	119.00	99.00
1.00	5	5.00	5.00	10.00	29.00	29.00	36.00	5.00	5.00	9.00	109.00	109.00	122.00
1.00	1	0.00	0.00	99.00	32.00	32.00	99.00	2.00	2.00	99.00	134.00	134.00	99.00
1.00	8	99.00	99.00	99.00	99.00	99.00	99.00	99.00	99.00	99.00	99.00	99.00	99.00
1.00	8	99.00	99.00	99.00	99.00	99.00	99.00	99.00	99.00	99.00	99.00	99.00	99.00
1.00	6	7.00	7.00	4.00	27.00	27.00	32.00	5.00	5.00	12.00	53.00	53.00	98.00
1.00	9	3.00	3.00	3.00	40.00	40.00	33.00	9.00	9.00	5.00	122.00	122.00	132.00
1.00	5	99.00	99.00	99.00	99.00	99.00	99.00	99.00	99.00	99.00	99.00	99.00	99.00
1.00	7	3.00	3.00	1.00	32.00	32.00	31.00	9.00	9.00	15.00	109.00	109.00	122.00
1.00	3	4.00	4.00	6.00	26.00	26.00	30.00	2.00	2.00	8.00	117.00	117.00	125.00
1.00	0	1.00	1.00	1.00	26.00	26.00	24.00	11.00	11.00	6.00	130.00	130.00	135.00
1.00	5	3.00	3.00	5.00	31.00	31.00	99.00	5.00	5.00	14.00	130.00	130.00	131.00
1.00	4	2.00	2.00	4.00	28.00	28.00	33.00	0.00	0.00	4.00	131.00	131.00	137.00
1.00	7	99.00	99.00	99.00	99.00	99.00	99.00	99.00	99.00	99.00	99.00	99.00	99.00
1.00	6	8.00	8.00	99.00	40.00	40.00	99.00	2.00	2.00	99.00	110.00	110.00	99.00
1.00	0	99.00	99.00	99.00	99.00	99.00	99.00	99.00	99.00	99.00	99.00	99.00	99.00
1.00	0	3.00	3.00	4.00	28.00	28.00	30.00	3.00	3.00	11.00	121.00	121.00	129.00
1.00	2	2.00	2.00	99.00	32.00	32.00	99.00	0.00	0.00	99.00	123.00	123.00	99.00
1.00	0	0.00	0.00	0.00	34.00	34.00	30.00	3.00	3.00	2.00	122.00	122.00	137.00
1.00	2	4.00	4.00	3.00	42.00	42.00	42.00	7.00	7.00	5.00	118.00	118.00	127.00
1.00	6	13.00	13.00	5.00	46.00	46.00	43.00	14.00	14.00	10.00	110.00	110.00	116.00
1.00	7	10.00	10.00	1.00	24.00	24.00	24.00	15.00	15.00	7.00	97.00	97.00	120.00

1.00	11	3.00	3.00	1.00	28.00	28.00	34.00	0.00	0.00	3.00	135.00	135.00	115.00
1.00	5	99.00	99.00	99.00	99.00	99.00	99.00	99.00	99.00	99.00	99.00	99.00	99.00
1.00	9	99.00	99.00	99.00	99.00	99.00	99.00	99.00	99.00	99.00	99.00	99.00	99.00
1.00	3	4.00	4.00	3.00	29.00	29.00	30.00	5.00	5.00	11.00	124.00	124.00	127.00
1.00	6	9.00	9.00	7.00	38.00	38.00	39.00	9.00	9.00	11.00	109.00	109.00	122.00
1.00	5	99.00	99.00	99.00	99.00	99.00	99.00	99.00	99.00	99.00	99.00	99.00	99.00
1.00	8	11.00	11.00	4.00	46.00	46.00	39.00	27.00	27.00	18.00	99.00	99.00	118.00
1.00	6	5.00	5.00	99.00	33.00	33.00	99.00	2.00	2.00	99.00	131.00	131.00	99.00
1.00	4	1.00	1.00	1.00	29.00	29.00	31.00	4.00	4.00	8.00	120.00	120.00	120.00
1.00	6	99.00	99.00	99.00	99.00	99.00	99.00	99.00	99.00	99.00	99.00	99.00	99.00
1.00	3	99.00	99.00	99.00	99.00	99.00	99.00	99.00	99.00	99.00	99.00	99.00	99.00
1.00	12	8.00	8.00	5.00	39.00	39.00	32.00	3.00	3.00	0.00	121.00	121.00	124.00
1.00	6	4.00	4.00	2.00	36.00	36.00	38.00	18.00	18.00	21.00	110.00	110.00	117.00
1.00	99	99.00	99.00	99.00	50.00	50.00	99.00	11.00	11.00	99.00	100.00	100.00	99.00
1.00	1	99.00	99.00	99.00	99.00	99.00	99.00	99.00	99.00	99.00	99.00	99.00	99.00
1.00	3	2.00	2.00	4.00	35.00	35.00	32.00	3.00	3.00	2.00	116.00	116.00	134.00
1.00	3	5.00	5.00	2.00	38.00	38.00	38.00	7.00	7.00	12.00	112.00	112.00	115.00
1.00	6	1.00	1.00	4.00	35.00	35.00	99.00	4.00	4.00	2.00	125.00	125.00	126.00
1.00	2	0.00	0.00	2.00	28.00	28.00	31.00	1.00	1.00	6.00	135.00	135.00	137.00
1.00	0	2.00	2.00	99.00	26.00	26.00	99.00	7.00	7.00	99.00	118.00	118.00	99.00
1.00	10	99.00	99.00	99.00	99.00	99.00	99.00	99.00	99.00	99.00	99.00	99.00	99.00
1.00	8	4.00	4.00	99.00	30.00	30.00	99.00	8.00	8.00	99.00	108.00	108.00	99.00
1.00	8	7.00	7.00	12.00	34.00	34.00	39.00	6.00	6.00	15.00	109.00	109.00	99.00
1.00	1	99.00	99.00	99.00	99.00	99.00	99.00	99.00	99.00	99.00	99.00	99.00	99.00
1.00	3	99.00	99.00	0.00	23.00	23.00	22.00	8.00	8.00	2.00	124.00	124.00	134.00
1.00	5	6.00	6.00	99.00	41.00	41.00	99.00	4.00	4.00	99.00	116.00	116.00	99.00
1.00	6	2.00	2.00	6.00	31.00	31.00	37.00	10.00	10.00	7.00	124.00	124.00	133.00
1.00	1	1.00	1.00	99.00	33.00	33.00	99.00	7.00	7.00	99.00	127.00	127.00	99.00
1.00	7	7.00	7.00	5.00	34.00	34.00	31.00	10.00	10.00	11.00	114.00	114.00	99.00
1.00	0	0.00	0.00	0.00	24.00	24.00	24.00	5.00	5.00	4.00	120.00	120.00	128.00
1.00	4	99.00	99.00	99.00	99.00	99.00	99.00	99.00	99.00	99.00	99.00	99.00	99.00
1.00	6	7.00	7.00	7.00	40.00	40.00	36.00	7.00	7.00	5.00	121.00	121.00	131.00
1.00	4	0.00	0.00	2.00	27.00	27.00	99.00	3.00	3.00	3.00	133.00	133.00	99.00

1.00	17	19.00	19.00	99.00	49.00	49.00	99.00	13.00	13.00	99.00	100.00	100.00	99.00
1.00	0	1.00	1.00	1.00	33.00	33.00	38.00	3.00	3.00	10.00	121.00	121.00	113.00
1.00	2	4.00	4.00	99.00	28.00	28.00	99.00	0.00	0.00	99.00	129.00	129.00	99.00
1.00	5	99.00	99.00	99.00	99.00	99.00	99.00	99.00	99.00	99.00	99.00	99.00	99.00
1.00	3	2.00	2.00	1.00	32.00	32.00	33.00	21.00	21.00	18.00	109.00	109.00	114.00
1.00	5	99.00	99.00	99.00	99.00	99.00	99.00	99.00	99.00	99.00	99.00	99.00	99.00
1.00	7	99.00	99.00	99.00	99.00	99.00	99.00	99.00	99.00	99.00	99.00	99.00	99.00
1.00	1	5.00	5.00	5.00	36.00	36.00	41.00	6.00	6.00	15.00	118.00	118.00	110.00
1.00	8	99.00	99.00	99.00	99.00	99.00	99.00	99.00	99.00	99.00	99.00	99.00	99.00
1.00	1	5.00	5.00	4.00	36.00	36.00	37.00	8.00	8.00	12.00	120.00	120.00	125.00
1.00	8	99.00	99.00	99.00	99.00	99.00	99.00	99.00	99.00	99.00	99.00	99.00	99.00
1.00	5	99.00	99.00	99.00	99.00	99.00	99.00	99.00	99.00	99.00	99.00	99.00	99.00
1.00	0	99.00	99.00	99.00	99.00	99.00	99.00	99.00	99.00	99.00	99.00	99.00	99.00
1.00	2	8.00	8.00	11.00	42.00	42.00	45.00	7.00	7.00	18.00	120.00	120.00	108.00
1.00	11	99.00	99.00	99.00	99.00	99.00	99.00	99.00	99.00	99.00	99.00	99.00	99.00
1.00	7	3.00	3.00	2.00	34.00	34.00	32.00	2.00	2.00	7.00	125.00	125.00	122.00
1.00	2	99.00	99.00	99.00	99.00	99.00	99.00	99.00	99.00	99.00	99.00	99.00	99.00
1.00	3	3.00	3.00	1.00	29.00	29.00	27.00	6.00	6.00	5.00	119.00	119.00	133.00
1.00	1	2.00	2.00	5.00	35.00	35.00	33.00	5.00	5.00	8.00	121.00	121.00	125.00
1.00	5	99.00	99.00	99.00	99.00	99.00	99.00	99.00	99.00	99.00	99.00	99.00	99.00
1.00	2	99.00	99.00	99.00	99.00	99.00	99.00	99.00	99.00	99.00	99.00	99.00	99.00
1.00	3	6.00	6.00	6.00	37.00	37.00	29.00	9.00	9.00	12.00	118.00	118.00	128.00
1.00	3	99.00	99.00	99.00	99.00	99.00	99.00	99.00	99.00	99.00	99.00	99.00	99.00
1.00	15	99.00	99.00	99.00	99.00	99.00	99.00	99.00	99.00	99.00	99.00	99.00	99.00
1.00	9	7.00	7.00	21.00	38.00	38.00	40.00	31.00	31.00	44.00	101.00	101.00	96.00
1.00	2	1.00	1.00	2.00	21.00	21.00	24.00	0.00	0.00	3.00	137.00	137.00	131.00
1.00	99	99.00	99.00	99.00	99.00	99.00	99.00	99.00	99.00	99.00	99.00	99.00	99.00
1.00	12	8.00	8.00	0.00	32.00	32.00	29.00	3.00	3.00	0.00	118.00	118.00	132.00
1.00	4	2.00	2.00	99.00	33.00	33.00	99.00	4.00	4.00	99.00	123.00	123.00	99.00
1.00	3	2.00	2.00	99.00	32.00	32.00	99.00	28.00	28.00	99.00	106.00	106.00	99.00
1.00	10	99.00	99.00	99.00	99.00	99.00	99.00	99.00	99.00	99.00	99.00	99.00	99.00
2.00	9999	6.00	3.00	2.00	31.00	31.00	29.00	14.00	6.00	9999.00	125.00	125.00	9999.00
2.00	9999	9999.00	9999.00	9999.00	9999.00	9999.00	9999.00	99.00	99.00	9999.00	9999.00	9999.00	9999.00

2.00	3	3.00	6.00	4.00	33.00	33.00	34.00	15.00	17.00	14.00	114.00	121.00	120.00
2.00	1	1.00	1.00	9999.00	31.00	9999.00	9999.00	14.00	99.00	9999.00	99.00	9999.00	9999.00
2.00	5	20.00	5.00	4.00	66.00	33.00	9999.00	22.00	6.00	8.00	97.00	125.00	129.00
2.00	4	4.00	2.00	9999.00	23.00	20.00	9999.00	8.00	4.00	9999.00	122.00	134.00	9999.00
2.00	0	9999.00	0.00	9999.00	9999.00	9999.00	9999.00	99.00	99.00	9999.00	9999.00	9999.00	999.00
2.00	10	9999.00	10.00	9999.00	65.00	9999.00	9999.00	99.00	99.00	9999.00	9999.00	9999.00	9999.00
2.00	2	7.00	3.00	2.00	40.00	41.00	37.00	7.00	8.00	9999.00	119.00	131.00	9999.00
2.00	12	13.00	0.00	5.00	37.00	32.00	39.00	1.00	1.00	4.00	134.00	139.00	142.00
2.00	0	1.00	0.00	0.00	22.00	22.00	20.00	4.00	2.00	9999.00	136.00	136.00	9999.00
2.00	0	2.00	0.00	9999.00	22.00	9999.00	9999.00	12.00	99.00	9999.00	141.00	9999.00	9999.00
2.00	9	8.00	9.00	9999.00	50.00	9999.00	9999.00	35.00	99.00	9999.00	78.00	9999.00	9999.00
2.00	1	12.00	4.00	11.00	29.00	28.00	9999.00	10.00	6.00	9999.00	119.00	119.00	9999.00
2.00	7	5.00	0.00	0.00	45.00	28.00	25.00	13.00	12.00	0.00	102.00	116.00	127.00
2.00	7	6.00	6.00	9999.00	39.00	9999.00	9999.00	14.00	19.00	9999.00	117.00	111.00	9999.00
2.00	1	1.00	1.00	9999.00	9999.00	9999.00	9999.00	99.00	99.00	9999.00	99.00	9999.00	9999.00
2.00	1	0.00	0.00	0.00	20.00	20.00	21.00	6.00	6.00	8.00	124.00	124.00	141.00
2.00	2	5.00	6.00	6.00	31.00	25.00	29.00	8.00	99.00	9999.00	120.00	9999.00	9999.00
2.00	3	4.00	0.00	9999.00	27.00	21.00	9999.00	2.00	2.00	9999.00	130.00	131.00	9999.00
2.00	1	6.00	3.00	13.00	41.00	9999.00	51.00	32.00	11.00	28.00	88.00	106.00	9999.00
2.00	6	11.00	11.00	9.00	39.00	46.00	44.00	23.00	30.00	20.00	102.00	85.00	108.00
2.00	14	13.00	14.00	9.00	57.00	57.00	9999.00	4.00	3.00	2.00	131.00	129.00	132.00
2.00	7	8.00	10.00	9.00	43.00	49.00	45.00	25.00	26.00	23.00	105.00	107.00	113.00
2.00	9	10.00	9.00	9999.00	34.00	24.00	9999.00	13.00	13.00	9999.00	127.00	130.00	9999.00
2.00	0	2.00	3.00	0.00	26.00	24.00	22.00	9.00	12.00	9999.00	123.00	127.00	9999.00
2.00	2	1.00	2.00	1.00	20.00	23.00	20.00	8.00	3.00	9999.00	143.00	144.00	9999.00
2.00	2	2.00	0.00	0.00	20.00	9999.00	21.00	3.00	0.00	9999.00	132.00	138.00	9999.00
2.00	13	9999.00	13.00	9999.00	9999.00	9999.00	9999.00	99.00	99.00	9999.00	9999.00	9999.00	9999.00
2.00	5	8.00	3.00	3.00	23.00	25.00	29.00	6.00	4.00	9999.00	128.00	114.00	9999.00
2.00	2	5.00	6.00	2.00	31.00	28.00	33.00	7.00	6.00	5.00	124.00	120.00	124.00
2.00	5	2.00	3.00	4.00	25.00	9999.00	32.00	6.00	11.00	5.00	121.00	121.00	118.00
2.00	9	5.00	2.00	3.00	30.00	28.00	30.00	14.00	99.00	9999.00	109.00	9999.00	9999.00
2.00	3	1.00	1.00	4.00	27.00	25.00	28.00	13.00	9.00	15.00	123.00	115.00	115.00
2.00	0	0.00	0.00	9999.00	9999.00	9999.00	9999.00	99.00	99.00	9999.00	9999.00	9999.00	9999.00

2.00	9999	9999.00	9999.00	9999.00	9999.00	9999.00	9999.00	99.00	99.00	9999.00	9999.00	9999.00	9999.00
2.00	10	3.00	6.00	2.00	29.00	25.00	25.00	12.00	1.00	1.00	130.00	139.00	140.00
2.00	9	9.00	6.00	3.00	32.00	28.00	27.00	15.00	13.00	17.00	121.00	119.00	117.00
2.00	5	17.00	9.00	9.00	60.00	51.00	40.00	36.00	20.00	9999.00	85.00	117.00	9999.00
2.00	6	11.00	11.00	8.00	40.00	36.00	38.00	14.00	18.00	9999.00	112.00	108.00	9999.00
2.00	1	9999.00	1.00	9999.00	31.00	9999.00	9999.00	99.00	99.00	9999.00	9999.00	9999.00	9999.00
2.00	11	2.00	4.00	3.00	34.00	25.00	28.00	99.00	17.00	4.00	109.00	125.00	122.00
2.00	4	7.00	6.00	7.00	37.00	31.00	38.00	9.00	3.00	4.00	124.00	123.00	126.00
2.00	1	9999.00	1.00	9999.00	9999.00	9999.00	9999.00	99.00	99.00	9999.00	99.00	9999.00	9999.00
2.00	10	9999.00	2.00	9999.00	28.00	9999.00	9999.00	12.00	99.00	12.00	133.00	9999.00	120.00
2.00	6	9999.00	6.00	9999.00	39.00	9999.00	9999.00	99.00	99.00	9999.00	9999.00	9999.00	9999.00
2.00	8	0.00	1.00	2.00	39.00	39.00	39.00	6.00	4.00	5.00	126.00	136.00	129.00
2.00	8	0.00	2.00	4.00	28.00	34.00	36.00	6.00	7.00	9.00	129.00	133.00	9999.00
2.00	2	4.00	0.00	0.00	31.00	29.00	30.00	28.00	4.00	3.00	99.00	135.00	135.00
2.00	5	11.00	6.00	12.00	36.00	41.00	55.00	18.00	25.00	28.00	116.00	105.00	101.00
2.00	5	12.00	5.00	9.00	49.00	9999.00	9999.00	27.00	99.00	29.00	107.00	9999.00	112.00
2.00	4	6.00	6.00	9999.00	27.00	28.00	9999.00	99.00	11.00	9999.00	9999.00	119.00	9999.00
2.00	0	1.00	0.00	0.00	23.00	23.00	26.00	13.00	4.00	1.00	137.00	136.00	141.00
2.00	2	3.00	3.00	3.00	29.00	40.00	42.00	11.00	1.00	15.00	125.00	131.00	113.00
2.00	6	10.00	8.00	4.00	26.00	30.00	29.00	10.00	99.00	9999.00	130.00	9999.00	9999.00
2.00	4	0.00	5.00	9999.00	25.00	30.00	9999.00	1.00	7.00	9999.00	9999.00	118.00	9999.00
2.00	0	0.00	1.00	0.00	24.00	22.00	22.00	11.00	1.00	1.00	129.00	135.00	144.00
2.00	9999	7.00	4.00	7.00	24.00	33.00	9999.00	99.00	99.00	19.00	121.00	126.00	9999.00
2.00	9	9.00	7.00	9999.00	43.00	44.00	9999.00	10.00	8.00	9999.00	96.00	115.00	9999.00
2.00	4	0.00	3.00	9999.00	25.00	25.00	9999.00	2.00	3.00	9999.00	137.00	134.00	9999.00
2.00	3	3.00	3.00	1.00	28.00	9999.00	26.00	5.00	2.00	5.00	123.00	118.00	121.00
2.00	3	1.00	3.00	3.00	22.00	9999.00	22.00	5.00	99.00	5.00	133.00	9999.00	134.00
2.00	0	5.00	4.00	6.00	24.00	37.00	34.00	14.00	12.00	9999.00	129.00	124.00	9999.00
2.00	9999	10.00	5.00	11.00	29.00	32.00	34.00	14.00	2.00	13.00	128.00	120.00	112.00
2.00	4	3.00	4.00	9999.00	24.00	9999.00	9999.00	0.00	99.00	9999.00	132.00	9999.00	9999.00
2.00	12	9999.00	12.00	9999.00	9999.00	9999.00	9999.00	99.00	99.00	9999.00	9999.00	9999.00	9999.00
2.00	7	9999.00	7.00	9999.00	9999.00	9999.00	9999.00	99.00	99.00	9999.00	9999.00	9999.00	9999.00
2.00	2	6.00	6.00	13.00	52.00	56.00	51.00	16.00	99.00	9999.00	96.00	9999.00	9999.00

2.00	0	1.00	3.00	0.00	26.00	29.00	9999.00	99.00	11.00	6.00	122.00	122.00	127.00
2.00	1	3.00	2.00	9999.00	27.00	24.00	9999.00	11.00	14.00	9999.00	122.00	131.00	9999.00
2.00	9999	9999.00	9999.00	9999.00	9999.00	9999.00	9999.00	99.00	99.00	9999.00	9999.00	9999.00	9999.00
2.00	1	10.00	5.00	11.00	43.00	47.00	48.00	11.00	10.00	9999.00	103.00	103.00	9999.00
2.00	10	7.00	5.00	17.00	47.00	41.00	62.00	99.00	99.00	9999.00	9999.00	9999.00	9999.00
2.00	2	9999.00	2.00	9999.00	9999.00	9999.00	9999.00	99.00	99.00	9999.00	9999.00	9999.00	9999.00
2.00	3	9999.00	3.00	9999.00	9999.00	9999.00	9999.00	99.00	99.00	9999.00	9999.00	9999.00	9999.00
2.00	7	7.00	7.00	9999.00	9999.00	9999.00	9999.00	99.00	10.00	9999.00	9999.00	9999.00	9999.00
2.00	5	4.00	5.00	0.00	32.00	9999.00	29.00	3.00	99.00	1.00	126.00	9999.00	137.00
2.00	5	8.00	5.00	4.00	26.00	9999.00	29.00	8.00	99.00	9999.00	107.00	9999.00	9999.00
2.00	2	2.00	0.00	9999.00	24.00	24.00	21.00	4.00	4.00	9999.00	136.00	127.00	9999.00
2.00	8	8.00	10.00	5.00	39.00	34.00	29.00	99.00	19.00	9999.00	106.00	106.00	9999.00
2.00	6	7.00	5.00	9999.00	36.00	34.00	9999.00	11.00	8.00	9999.00	118.00	128.00	9999.00
2.00	0	0.00	2.00	1.00	27.00	31.00	25.00	99.00	1.00	9999.00	133.00	130.00	9999.00
2.00	3	4.00	5.00	4.00	28.00	28.00	28.00	4.00	4.00	9.00	120.00	120.00	9999.00
2.00	0	9999.00	0.00	9999.00	9999.00	9999.00	9999.00	99.00	99.00	9999.00	9999.00	9999.00	9999.00
