# Supplementary material for: The impact of the early COVID-19 pandemic on maternal mental health during pregnancy and postpartum
Source: PLoS One. 2024 Sep 20;19(9):e0310902. doi: 10.1371/journal.pone.0310902 (PMC11414898; doi:10.1371/journal.pone.0310902)
Supplement: Attachment — Submitted filename: REVIEWERS COMMENTS.docx [file pone.0310902.s002.docx]

REVIEWER 1

Introduction:
line 64, please rephrase line 64, in the first sentence to enhance understandability.
line 74: state full meaning of NICU .

Method:
State the means of the postpartum follow-ups, virtually or physically or mixed?
Reflect the sample size(s).

Result:

line 240-- grammar check. ... but not was not...

Conclusion:
Line 329, ... but could negatively and positively influence individual aspects. OF WHAT?
You mean aspects of self-confidence and bonding respectively? Please clarify.

Reference:
Please, manually edit and format most of the references in the reference page.


General manuscript presentation not well structured. E.g Seeing result table in the Methodology subsection.

REVIEWER 2

The manuscript should be impersonal. It is a manuscript not a descriptive essay as the authors depicted in some parts of the manuscript. All pronouns such as, 'we' and our should be removed and replaced appropriately. This was shown in sections such as the abstract, last paragraph in introduction session, Post partum bonding, conclusion etc.
