# Supplementary material for: The impact of the early COVID-19 pandemic on maternal mental health during pregnancy and postpartum
Source: PLoS One. 2024 Sep 20;19(9):e0310902. doi: 10.1371/journal.pone.0310902 (PMC11414898; doi:10.1371/journal.pone.0310902)
Supplement: Attachment — Submitted filename: Response to Reviewers_R1_end.docx [file pone.0310902.s003.docx]

**Response to Revieweers:**

Reviewer #1: Introduction:
line 64, please rephrase line 64, in the first sentence to enhance understandability.

Many thanks for this hint. We have rephrased the two sentences as suggested.

line 74: state full meaning of NICU .

NICU means “neonatal intensive care unit”. We noticed by your comment that we had written “NICU” instead of “ICU” (intensive care unit). Since this sentence is about maternal risks, ICU is the correct term and we have explained the abbreviation.

Method:
State the means of the postpartum follow-ups, virtually or physically or mixed?

Thank you, we have added the requested information about the postpartum follow-ups in the methods section.

Reflect the sample size(s).

We have added the respected sample sized in the method section for clarification.

Result:

line 240-- grammar check. ... but not was not...

Thanks, done.

Conclusion:
Line 329, ... but could negatively and positively influence individual aspects. OF WHAT?
You mean aspects of self-confidence and bonding respectively? Please clarify.

Thank you, we have clarified it in the revised conclusion.

Reference:
Please, manually edit and format most of the references in the reference page.

We have reviewed our reference list and corrected reference 35 and 37:

- [35] Spielberger CD, Gorsuch RL, Lushene RE. Manual for the State-Trait Anxiety Inventory. Palo Alto, CA, 1970: Consulting Psychologists Press
- [37] Laux L, Glanzmann P, Schaffner P, Spielberger CD. Das State-Trait-Angstinventar. Theoretische Grundlagen und Handanweisung. Weinheim, 1981: Beltz Test GmbH

There was a rather minor erratum of the references which we have included in the reference list. We therefore have two more literature sources.

- [9] Villar et al. 2021: Misspelled Author Name in the Byline. JAMA Pediatr. 2022 Jan 1;176(1):104. doi: 10.1001/jamapediatrics.2021.4953. Erratum for: JAMA Pediatr. 2021 Aug 1;175(8):817-826. PMID: 34779828; PMCID: PMC8593825.
- [47] Lebel et al. 2020: Lebel C, MacKinnon A, Bagshawe M, Tomfohr-Madsen L, Giesbrecht G. Corrigendum to elevated depression and anxiety symptoms among pregnant individuals during the COVID-19 pandemic journal of affective disorders 277 (2020) 5-13. J Affect Disord. 2021 Jan 15;279:377-379. doi: 10.1016/j.jad.2020.10.012. Epub 2020 Nov 18. Erratum for: J Affect Disord. 2020 Dec 1;277:5-13. PMID: 33099052; PMCID: PMC8445275.

General manuscript presentation not well structured. E.g Seeing result table in the Methodology subsection.

Many thanks for this hint. We have included Table 1 in the results section and carefully checked the rest of the structure of the manuscript.

Reviewer #2: The manuscript should be impersonal. It is a manuscript not a descriptive essay as the authors depicted in some parts of the manuscript. All pronouns such as, 'we' and our should be removed and replaced appropriately. This was shown in sections such as the abstract, last paragraph in introduction session, Post partum bonding, conclusion etc.

Thank you. We replaced the personal pronouns in whole manuscript.
